# Micro-CT Evaluation of Gutta-Percha Removal by Two Retreatment Systems

**DOI:** 10.22037/iej.v13i2.18599

**Published:** 2018

**Authors:** Gustavo Alberto Rubino, George Táccio de Miranda Candeiro, Laila Gonzales Freire, Elaine Faga Iglecias, Érico de Mello Lemos, Celso Luiz Caldeira, Giulio Gavini

**Affiliations:** a *Faculdade de Odontologia, Post-graduate Program in Endodontics, University of São Paulo, São Paulo, Brazil; *; b * Centro Universitário Christus, Curso de Odontologia, Fortaleza, CE, Brazil; *; c * Universidade Santa Cecília, Curso de Odontologia, Discipline of Endodontics, Santos, SP, Brazil; *; d * Faculdade de Odontologia, Discipline of Endodontics, Department of Restorative Dentistry, University of São Paulo, São Paulo, SP, Brazil*

**Keywords:** Dental Instruments, Endodontics, Endodontic Retreatment, Gutta-Percha

## Abstract

**Introduction:**

The aim of the present *ex vivo* research was to compare the remaining filling material and the volumes of dentine removed after retreatment of curved canals with two rotary systems naming ProTaper Universal Retreatment and Mani NRT-GPR using micro-computed tomography (micro-CT).

**Methods and Material:**

Forty mandibular molars containing two completely separated canals, with curvature angle of 25-35^°^ and a curvature radius <10 mm were prepared to the Mtwo instrument 35/0.04 and filled with warm gutta-percha and AH-Plus sealer. The teeth were randomly divided into 2 groups (*n*=20), according to the retreatment system evaluated: ProTaper Universal Retreatment (PR group) or Mani NRT-GPR (MR group). Retreatment was considered complete when the working length was reached and when smooth dentinal walls were observed, with no evidence of filling material adhered to instruments or in the irrigating solution. Preoperative and postoperative micro-CT images were obtained with an isotropic voxel size of 11.88 µm to observe the volume of residual filling material in the canals and dentine removed after retreatment. Statistical analysis was performed by Student’s *t*-test (*P*<0.05).

**Results:**

The mean percentage of remaining filling material was 12.96% for PR group and 24.26% for MR group (*P*=0.0056). The percentage of dentin removal was greater in the PR group (5.02%) than MR group (1.36%) (*P*=0.0028). Both systems failed to completely remove the filling material from the canals.

**Conclusion:**

ProTaper Universal Retreatment system was more effective than Mani NRT-GPR system regarding removal of root filling material and also removed significantly more dentine after retreatment of curved mesial root canals of mandibular molars.

## Introduction

The primary goal of non-surgical endodontic retreatment is to achieve access to the root canal system by complete removal of previous filling material, followed by canal cleaning, shaping and refilling. Nevertheless, substantial amounts of filling material commonly remain in the canal after retreatment procedures [1-3].

Different techniques have been proposed to remove gutta-percha and sealer from root canals, including the use of hand files [1, 4], nickel-titanium (NiTi) rotary instruments [5], and NiTi systems with reciprocating [6, 7] or adaptive motion [8]. It has been shown that automated NiTi instruments allow for safer and faster removal of filling material compared to manual techniques [2, 9]. Some NiTi systems specifically designed for retreatment purposes have physical characteristics such as cross-sectional design, cutting angle, and presence/absence of an active tip that may affect gutta-percha and dentin removal from the root canal [4, 8-10].

The ProTaper Universal Retreatment files (Dentsply Maillefer, Ballaigues, Switzerland) have been evaluated in terms of their efficacy, cleaning ability and safety, either in straight or in curved root canals [2, 5, 9-12]. The Mani NRT-GPR gutta-percha removal system (Mani Inc., Utsunomiya, Japan) was introduced specifically for retreatment purposes, consisting of four instruments: two made of stainless steel to act in the upper portions of the root canal, and two made of NiTi, for the apical thirds of the canal. Up to the moment, limited data on the effectiveness of Mani NRT-GPR system is available.

In order to evaluate effectiveness of endodontic retreatment, several experimental models are used; however most involve transverse or longitudinal sections of the roots [7, 8, 10], analysis of radiographic images or clearing the tooth [1, 2], making impossible to quantify the volume of residual filling materials. 

Contrastingly, micro-computed tomography (micro-CT) is a non-invasive technique that enables three-dimensional quantitative evaluation of filling material and the amount of dentin removed during retreatment [6, 9, 13, 14].

The aim of the present study was to compare the remnants of root filling material and the volumes of dentine removed after the retreatment of curved mesial canals of mandibular molars, using two rotary systems, ProTaper Universal Retreatment (PR) and Mani NRT-GPR (MR), by means of micro-CT. The null hypothesis tested was that there is no difference in the performance of the different rotary retreatment systems in the removal of filling material and dentin from the root canal.

## Materials and Methods

Firstly, Ethics Committee in Research from Faculty of Dentistry of University of São Paulo approved the present research, under protocol number CAAE 0148.0.017.000-11.

A total of 40 human mandibular molars recently extracted were selected and stored in purified filtered water. The inclusion criteria involved teeth that presented fully formed apices, mesial roots with two completely separated canals from the orifice to the apex, a curvature angle of 25-35 degrees, according to Schneider’s technique [15] and a curvature radius less than 10 mm. Teeth that exhibited pulp calcifications, root resorption, previous endodontic treatment or root fractures were excluded. In order to standardize the specimens, all teeth were previously scanned by SkyScan 1172 micro-CT scanner (Bruker-microCT, Kontich, Belgium), that allows for scanning of high-density objects, with an isotropic voxel size of 11.88 μm and a copper-aluminum filter. Other parameters included x-ray voltage of 100 kV (10 W and 100 μA), 1475 ms exposure time, 360^°^ rotation, and 0.4^°^ rotation step. Only teeth with Vertucci’s classification type-II were selected in the present study [16]. 

***Root canal preparation***

Access cavities were prepared using round diamond burs mounted in a high-speed handpiece. Initial exploration of the mesial canals was carried out using a #10 K-type hand file (Dentsply Maillefer, Ballaigues, Switzerland). Root canals in which a #15 K-file (Dentsply Maillefer, Ballaigues, Switzerland) was loose were also excluded from the sample. The working length was determined 1 mm shorter of the point where the tip of the instrument became visible at the apical foramen, with the aid of an optical microscope (Alliance, São Carlos, Brazil). 

The canals were prepared using the Mtwo system (VDW, Munich, Germany) NiTi rotary files, starting with file 10/0.04, and then followed by 15/0.05, 20/0.06, 25/0.06, 30/0.05, and 35/0.04. Rotary files were operated by a Root ZX II electric motor (J. Morita, Osaka, Japan) at a constant speed of 280 rpm, according to manufacturer’s recommendations. Between each file, the root canals were rinsed with 2 mL of 1% sodium hypochlorite (Fórmula & Ação, São Paulo, Brazil) delivered by a syringe and a 30-gauge needle (NaviTip, Ultradent, South Jordan, UT, USA). After completion of preparation, the root canals were irrigated with a final sequence of 6 mL of 17% EDTA (Formula & Ação, São Paulo, Brazil), for 3 min, and 6 mL of NaOCl and then dried with paper points (VDW, Munich, Germany).

***Obturation***

Obturation of mesiobuccal and mesiolingual canals was performed by continuous wave of condensation technique with FM gutta-percha cones (Odous de Deus, Belo Horizonte, Brazil) and AH-Plus root canal sealer (Dentsply De Trey Gmbh, Konstanz, Germany). The Fine Medium plugger of the E&Q Plus Duo Heat System (Meta Biomed Co., Chungbuk, Republic of Korea), set at 200^°^C, was introduced into the canal 3 mm short of working length. A backfill procedure was accomplished using the gun of the E&Q Plus Duo Heat System by plasticizing gutta-percha pellets at 170^°^C. Warm gutta-percha was inserted into the root canal at 2 mm increments and condensed using a plugger until the root canal was completely filled. The quality of root canal obturation were confirmed radiographically. Access cavities were temporarily sealed with Citodur (Wilkos, Rio de Janeiro, Brazil), and specimens were stored for 14 days at 37^°^ C and 100% humidity, to allow the sealer to set to the maximum extent. All endodontic procedures were performed by a single operator.

***Micro-CT scanning of previous retreatment***

Teeth were scanned after obturation and after retreatment, using a SkyScan 1172 micro-CT scanner (Bruker-microCT, Kontich, Belgium) that allows for scanning of high-density objects, with an isotropic voxel size of 11.88 μm and a copper-aluminum filter. Other parameters included x-ray voltage of 100 kV (10 W and 100 μA), 1475 ms exposure time, 360^°^ rotation, and 0.4^°^ rotation step. 

The images were reconstructed with NRecon v.1.6.9 software (Bruker-microCT, Kontich, Belgium) by using the modified Feldkamp cone-beam reconstruction algorithm. The original gray scale images were processed for noise reduction, with the fine-tuning function: gaussian filter (Smoothing, kernel=2), beam hardening correction of 40%, post-alignment of 0.50 to compensate possible misalignment during acquisition, and ring artifact correction of 10. 

***Retreatment procedure***

The teeth were randomly divided into 2 groups, according to the retreatment system evaluated. Both mesial canals were retreated using the same system: Mani NRT-GPR (MR group; *n*=20) or ProTaper Universal Retreatment (PR group; *n*=20). In the PR group, ProTaper Universal Retreatment files D1, D2, and D3 were used in a crown-down sequence. Instrument D1 (30/0.09) was used to remove the filling material from the coronal third of the root canal, followed by D2 (25/0.08) in the middle third, and finally D3 (20/0.07) used to working length. Instruments were operated at 500 rpm and no torque control, as recommended by the manufacturer. In the MR group, the first instrument was 1S (70/0.04), used only in the canal entrance. File 2S (50/0.04) was used in the coronal third of the canals, followed by 3N (40/0.04) in the middle third, and 4N (30/0.04) in the apical third. These instruments were operated at 1000 rpm and no torque control, according to manufacturer’s recommendations. In both experimental groups, instrument penetration was carried out with light apical pressure. Retreatment was considered concluded when the working length was reached and smooth dentin walls were observed, with no evidence of filling material on dentinal walls, on the instruments or in the irrigation solution when observed under optical microscopy. During retreatment, canals were constantly irrigated with 1% sodium hypochlorite. After using the last file in each system, canals were once again irrigated with 10 mL of 1% sodium hypochlorite and dried using paper points. Every file, regardless of retreatment system, was discarded after four uses or whenever the threads showed any visible damage. Each root was then scanned again by using the SkyScan 1172 scanner with the same parameters.

***Micro-CT measurements and evaluations***

The resulting images from preoperative and postoperative scans were geometrically aligned using the 3D registration function of the DataViewer v.1.5.1 software (Bruker micro-CT, Bruker Corp. Billerica, MA, USA). The volume of interest for each specimen, extending from the furcation region to the apex of the mesial root, was set by integration of the regions of interest in all cross sections. The image datasets were processed with the CTAn v.1.14.4 software (Bruker micro-CT, Bruker Corp. Billerica, MA, USA), where task lists were applied to create separated binary images of the filling material and the dentin, by using a custom-processing tool with functions and mathematical operations. 

The grayscale range required to recognize each object to be evaluated in a density histogram was determined (thresholding), generating images comprising black pixels that represented voids (air) or white pixels representing the object of interest. Because filling material and dentin have significant different threshold values, volumetric analysis and 3D models were performed. 

The percentage of remaining filling material in the mesiobuccal and mesiolingual canals after retreatment and the percentage of dentin removed during retreatment were calculated as follows:

A/B×100= Volume (%) of remaining filling material

(C-D)/E×100= Volume (%) of dentin removed 

Where A represents Final volume of filling material, B; Initial volume of filling material, C; Initial volume of dentin, D; Final volume of remaining dentin and E stands for Initial volume of dentin. 

**Table 1:** Mean initial and final volumes of filling material (mm^3^), mean percentages of remaining filling material (%), and standard deviations

**Group**	**Mean initial volume **	**Mean final volume **	**Mean percentage (%)**	***P*** **-value**
**PR**	15.73	1.91	12.96 (6.77)	0.0056
**MR**	15.05	3.43	24.26 (9.99)	

*PR=ProTaper Retreatment; MR=Mani Retreatment*

**Table 2:** Mean initial and final volumes of dentin (mm3), mean percentages of dentin removal after retreatment (%), and standard deviations

**Group**	**Mean initial volume **	**Mean final volume **	**Mean percentage (%)**	***P*** **-value**
**PR**	129.44	122.81	5.02 (2.74)	0.0028
**MR**	129.24	127.48	1.36 (1.48)	

*PR=ProTaper Retreatment; MR=Mani Retreatment*

**Figure 1:**
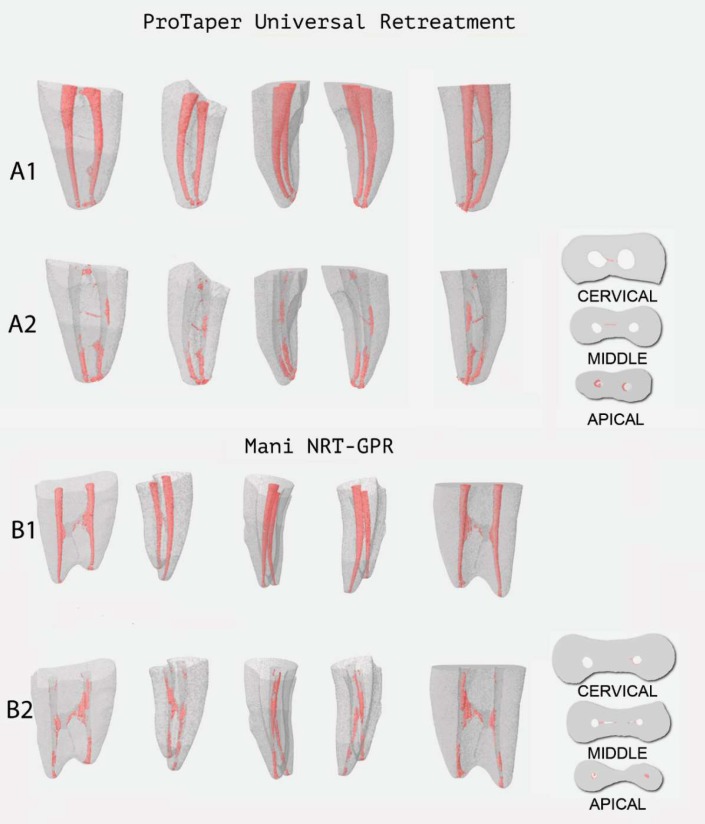
Analysis of removal procedure by micro-CT in mesial canals of mandibular molars with nickel-titanium rotary instruments. (A1/B1) Before, (A2/B2) superimposed images after the removal procedure

***Statistical analysis***

Statistical analyses were performed with GraphPad Prism 5.03 software (GraphPad Software, Inc., La Jolla, CA, USA). Since the study included two independent samples with normal distribution and homogeneity of variance, Student’s* t-*test was used, at the 95% confidence level (*P*<0.05), to determine the presence of statistical differences between the experimental groups.

## Results

Remaining filling material was observed in all specimens from both experimental groups, mainly in apical third (Figure 1). Table 1 shows mean initial and final volumes of filling material and mean percentages of remaining filling material.

The PR group showed statistically lower mean percentage of remaining filling material than MR group (*P*=0.0056). Nevertheless, PR group presented greater perceptual of dentin removal after retreatment than MR group: 5.02% and 1.36% respectively (*P*=0.0028) (Table 2).

No complications, such as creation of ledges, obstructions, perforations or instrument fractures were registered for the PR or MR instruments, after retreatment procedures. 

## Discussion

Complete removal of gutta-percha is a challenge for all current retreatment techniques, and the remaining material may decrease the effectiveness of disinfection procedures, such as additional instrumentation, activity of irrigating solutions and inter-appointment medication [17, 18]. 

In the present study, none of the retreatment techniques allowed for complete removal of filling material, which is consistent with the literature, where residual filling material was found in 100% of the samples [11, 12, 19]. The PR group exhibited statistically lower percentage of remaining filling material when compared with the MR group. In relation to the mean percentage of dentin removal, the PR group showed a 3.5-fold higher result. This finding rejects the null hypothesis, as the characteristics of different rotary retreatment systems seem to significantly affect filling material and dentin removal. 

Different methods have been used to assess removal of residual filling material from root canals after endodontic retreatment [2, 4, 7, 20]. Notwithstanding, studies based on 2-dimensional assessments, that evaluate only selected sections of the canal should be interpreted with caution, because results may vary among different observers, and loss of residual filling material may occur during splitting of specimens [1, 5, 21]. 

More recently, advanced applications of micro-CT scanning have emerged as a preferred method, providing high-resolution 3D volumetric data suitable for analysis, quantification and visualization of results [8, 12]. This non-destructive technique has been successfully used to evaluate the amount of filling material present in the root canal [13, 14], the volume of dentin removed during initial preparation [10, 18], the remaining filling material and dentin removed after retreatment [3, 6, 9].

Some limitations of this technique are related to methodological issues such as dependence of adequate training and hardware/software abilities. Successful high-density tissues imaging with micro-CT requires flexibility to alter the x-ray energy by selecting the correct voltage/filter, and appropriate processing of the original grayscale images for noise reduction, such as beam hardening correction [14, 21]. In this study, scanning was performed with an optimal resolution of 11.88 μm, over 360 degrees, with a high-applied x-ray voltage, a strong filter containing copper, and multiple frame averaging at each rotation step, allowing for analysis of the dentin close to the filling material, without artifacts [9].

Filling material can be removed from root canals using different approaches; however, mechanical techniques are increasingly adopted, due to the greater ability of NiTi instruments to remain centered during canal preparation and the shorter working time [2, 4, 5, 9]. In the present study, NiTi rotary ProTaper Universal Retreatment and Mani NRT-GPR systems were compared in terms of their effectiveness in removing gutta-percha from curved canals. Recently, it was observed no significant differences on apical debris extruded between ProTaper Universal Retreatment system and Mani NRT-GPR during retreatment procedure [22].

ProTaper Universal Retreatment instruments present a convex triangular cross-section with an internal angle of approximately 60^°^ at the cutting edge, increasing their capacity to remove filling material and consequently leading to dentin removal [5, 6]. Mani NRT-GPR instruments, in turn, were designed based on GPX stainless steel instruments (Brasseler, Savannah, GA, USA). They have a wide radial land and a neutral cutting angle, which reduces their cutting capacity and screw effect, but increases abrasion, justifying the manufacturer’s recommendations to use without torque control. NRT-GPR gutta-percha removal file (MANI, Utsunomiya, Japan) has recently begun selling that is different from those of other companies in that it rotates faster (1000 min), and that it is available in stainless steel for removing gutta-percha from the cervical third of the root canal, and in NiTi files for removing it from the periapical area or curved parts of the canal. This new file has only 1 cutting edge and one groove, while the ProTaper Universal retreatment file and Mtwo retreatment file have 3 and 2 cutting edges, respectively. The cutting edge of the NRT-GPR is R-Phase NiTi, which is different from the general superelastic NiTi files and becomes permanently distorted to a certain degree in accordance with the curvature of the root canal. In the present study, no files presented deformation or fracture, evidencing a suitable performance of this instrument. 

Another important aspect to consider when analyzing our results is the taper. All instruments of the ProTaper Universal Retreatment system showed tip diameters equal to or lower than those of the Mani NRT-GPR system, but their tapers were significantly higher, increasing the area of contact between the instrument and both the material filling and dentinal walls. 

The presence of remaining filling material in the apical third of all specimens may be justified by the fact that the last instrument used in both retreatment systems, ProTaper D3 (20/0.07) and Mani N4 (30/0.04), has a smaller tip size than the last instrument used during root canal preparation (Mtwo file 35/0.04). In addition, the extrusion of the filling material into the apical third and the complex anatomy of mesial canals of mandibular molars [1,4], especially in the apical third, make these areas challenging to retreat, allowing for a greater mean percentage of residual filling material [3, 8, 23].

The use of the continuous wave of condensation technique during obturation still may explain the persistence of remaining filling material in other areas of the root canal system [23, 24]. Warm gutta-percha is easily compacted into anatomical areas inaccessible to the instruments used during retreatment [13]. This may account for the greater percentage of remaining filling material found in this study than in previous ones employing cold obturation techniques [9, 11, 19, 20]. 

It remains clear that, in order to decrease the amount of remaining filling material after non-surgical retreatment, a new chemical-mechanical preparation of the root canal is necessary. The use of at least two diameters greater than the last instrument previously used, seems to represent an adequate balance between apical enlargement and conservation of tooth structure [10, 11, 18]. Nevertheless, during root canal retreatment, excessive removal of dentin should be avoided, in order to decrease the risk of perforations, cracks and vertical fractures [6]. In the present study, no complications, such as creation of ledges, obstructions, perforations, or instrument fractures, were observed.

In clinical practice, the removal of filling materials during retreatment can be completed with different techniques, including radiographs and microscope images, but also paper points soaked in solvent or a simple visual and tactile exam of the instrument. In the present study, as in others, retreatment was considered to be completed when remnants of filling material were no longer found on the latest instrument of each system or in the irrigation solution, and when smooth dentinal walls were observed and the working length was achieved [5, 10]. 

Because of the positive cutting angle and increased taper, the ProTaper Universal Retreatment system was effective in removing filling material, but also caused greater dentin removal. Thus, an instrument with its cross-sectional characteristic, presenting medium taper (0.04 or 0.05) and a great diameter tip may produce greater and safer gutta-percha removal.

Additional techniques to improve the removal of filling remnants are attempted as the use of ultrasonic agitation, lasers and XP-Endo Finisher files [3, 24, 25]. The motion of the NiTi instruments applied, under continuous rotation, reciprocation or the combination of both, is also an important factor in the removal of root fillings [7, 8]. Further studies should be performed in order to evaluate the effect of instrument movement and supplementary approaches to optimize endodontic retreatment.

## Conclusion

In conclusion, it was observed that, despite both instruments failed to remove completely filling material, ProTaper Universal Rotary Retreatment system was significantly more effective than Mani NRT-GPR system regarding removal of root filling material and also removed significantly more dentin after retreatment of curved mesial root canals of mandibular molars.
